# Considering the base rates of low performance in cognitively healthy older adults improves the accuracy to identify neurocognitive impairment with the Consortium to Establish a Registry for Alzheimer’s Disease-Neuropsychological Assessment Battery (CERAD-NAB)

**DOI:** 10.1007/s00406-014-0571-z

**Published:** 2015-01-03

**Authors:** Panagiota Mistridis, Simone C. Egli, Grant L. Iverson, Manfred Berres, Klaus Willmes, Kathleen A. Welsh-Bohmer, Andreas U. Monsch

**Affiliations:** 1 Memory Clinic, Felix Platter Hospital, University Center for Medicine of Aging Basel, Schanzenstrasse 55, 4031 Basel, Switzerland; 2 Department of Psychology, University of Basel, Missionsstrasse 60/62, 4055 Basel, Switzerland; 3 Department of Physical Medicine and Rehabilitation, Harvard Medical School, Boston, MA 02114 USA; 4 Red Sox Foundation and Massachusetts General Hospital Home Base Program, Boston, MA 02114 USA; 5 Department of Mathematics and Technology, University of Applied Sciences Koblenz, Joseph-Rovan-Allee 2, 53424 Remagen, Germany; 6 Section Neuropsychology, Department of Neurology, RWTH Aachen University, Pauwelsstraße 30, 52074 Aachen, Germany; 7 Joseph and Kathleen Bryan Alzheimer’s Disease Center, Duke University, 2200W Main Street, Suite A200, Durham, NC 27705 USA

**Keywords:** Neuropsychology, Normal aging, Diagnosis, Neurocognitive disorders, Dementia, Mild cognitive impairment

## Abstract

It is common for some healthy older adults to obtain low test scores when a battery of neuropsychological tests is administered, which increases the risk of the clinician misdiagnosing cognitive impairment. Thus, base rates of healthy individuals’ low scores are required to more accurately interpret neuropsychological results. At present, this information is not available for the German version of the Consortium to Establish a Registry for Alzheimer’s Disease-Neuropsychological Assessment Battery (CERAD-NAB), a frequently used battery in the USA and in German-speaking Europe. This study aimed to determine the base rates of low scores for the CERAD-NAB and to tabulate a summary figure of cut-off scores and numbers of low scores to aid in clinical decision making. The base rates of low scores on the ten German CERAD-NAB subscores were calculated from the German CERAD-NAB normative sample (*N* = 1,081) using six different cut-off scores (i.e., 1st, 2.5th, 7th, 10th, 16th, and 25th percentile). Results indicate that high percentages of one or more “abnormal” scores were obtained, irrespective of the cut-off criterion. For example, 60.6 % of the normative sample obtained one or more scores at or below the 10th percentile. These findings illustrate the importance of considering the prevalence of low scores in healthy individuals. The summary figure of CERAD-NAB base rates is an important supplement for test interpretation and can be used to improve the diagnostic accuracy of neurocognitive disorders.

**Electronic supplementary material:**

The online version of this article (doi:10.1007/s00406-014-0571-z) contains supplementary material, which is available to authorized users.

## Introduction

When administered a battery of neuropsychological tests, some healthy older adults obtain low scores (e.g., [1–6]). Low test scores, especially low memory scores, obtained by healthy older adults might be interpreted as an indication of cognitive deterioration and lead to a false-positive diagnosis of mild cognitive impairment (MCI). Thus, to evaluate test results accurately and safeguard against misinterpretation of abnormal test performance, knowledge about base rates of low scores in healthy older adults compared to true pathological performance is of critical importance.

Cognitive deficits, particularly in episodic memory, represent a hallmark of Alzheimer’s disease (AD) dementia and are known to be detectable in prodromal stages, such as MCI [7–9]. However, given that healthy older adults may also show a gradual but clinically insignificant cognitive decline over time [10, 11], these cognitive changes may be very subtle, such that in clinical practice detecting true cognitive impairment remains challenging [8]. Thus, to obtain an accurate understanding of an individual’s cognitive functioning for a reliable and valid diagnosis, it is very important to differentiate normal changes with cognitive aging from cognitive changes that go beyond normal aging.

At present, however, no universally accepted and empirically validated psychometric criteria exist to define cognitive impairment [12, 13]. Cognitive deterioration is broadly defined in clinical practice, and different cut-off scores are used to define impairment. Thus, when assessing cognitive impairment, some methodological issues have to be considered. When administering only one neuropsychological test, the number of individuals being within the lower tail of the Gaussian distribution will depend on the chosen cut-off score (e.g., when using the 7th percentile as the critical cut-off score, by definition, 7 % of cognitively healthy individuals would be erroneously classified as impaired). However, clinicians usually do not rely on a single test score, but on the patients’ performance on multiple tests when assessing cognition. The prevalence of low scores will then be considerably higher when the number of tests administered increases, compared to single test interpretation [1, 12]. Additionally, tests in a neuropsychological battery commonly show substantial intercorrelations and are therefore not independent, such that a non-consideration of this fact results in inaccurate assumptions (i.e., over- or underestimation) of neuropsychological test results. Thus, the number of tests in a test battery and the cut-off criterion applied affect the number of low scores: that is, as more tests are administered and less stringent [e.g., 25th percentile (*z* = −0.67)] cut-off scores are applied, then more “abnormal” scores will be obtained by healthy individuals [12, 14, 15]. Although the recommended criterion for low performance associated with MCI is 1.5 standard deviations (SD) below the mean of adjusted normative data [16], other cut-off scores ranging from 1.0 to 2.0 SD below the mean have been proposed by the National Institute on Aging and the Alzheimer’s Association [17] or the fifth edition of the American Psychiatric Association’s Diagnostic and Statistical Manual of Mental Disorders (DSM-5; [18]). However, the applied cut-off score needs to be considered carefully because the cut-off score selected substantially affects the rate of false-positive low scores [12, 19]. In addition, beside such psychometrical arguments, personal or situational factors may affect the prevalence of low scores (see [20]). For example, some people will be located at the lowest (and highest) tail of the Gaussian distribution, i.e., these individuals have always performed low in the past and will also show weak performances in the future, although they are healthy. Longstanding weaknesses in certain cognitive areas may also account for low scores as well as situational factors such as the personal (daily) condition (poor sleep quality, fatigue, low motivation, attentional distraction, discomfort during testing or tension, suffering from pain, bad emotional status, difficulties to adapt to test situation, etc.). Another source might consist of measurement errors such as misunderstandings of test instructions.

The presence of low scores in healthy older adults is a well-known phenomenon and a relevant issue for clinicians and researchers to consider, especially in order to safeguard against misinterpretation of some “abnormal” scores. However, this concept still remains relatively poorly understood and has certainly not yet been implemented in its full significance in everyday clinical practice. Low scores are a common feature in any battery of tests and have already been reported by several research groups for different neuropsychological tests or test batteries (e.g., [2]). However, at present this information is not available for the German version of the Consortium to Establish a Registry for Alzheimer’s Disease-Neuropsychological Assessment Battery (CERAD-NAB), a well-established and very commonly used battery in German-speaking Europe and the USA to assess mature individuals with potential neurocognitive disorders [21, 22]. The CERAD-NAB yields ten standardized *z*-scores and is useful in discriminating between healthy older adults and patients with incipient AD or AD dementia [21, 23, 24]. Incorporating the base rates of low scores into clinical practice might reduce false-positive diagnoses that may cause anxiety and distress in both the affected individuals and their families, as well as reduce false-negative diagnoses that would prevent patients from receiving and profiting from early therapeutic interventions.

The objective of the present study was to determine base rates of low cognitive scores in healthy older adults on the CERAD-NAB using empirical data from an assessment of a large normative sample of healthy older participants. To account for the well-known influence of demographic variables, we used standardized *z*-scores adjusted for age, gender, and education [25] in all analyses. The base rates of low scores in the normative sample were calculated for multiple cut-off scores for the entire battery. In a further step, we provide a summary figure that tabulates the number of low CERAD scores for six cut-off scores (i.e., 1st, 2.5th, 7th, 10th, 16th, and 25th percentile) to aid clinicians to diagnose and differentiate between normal and abnormal performance. To examine the usefulness of this summary figure in identifying individuals at risk for cognitive deterioration at a very early stage, baseline performance of initially healthy participants who developed AD dementia several years later was compared with participants who remained healthy.

## Methods

### Participants

The study sample consisted of 1,099 healthy older participants from the BASEL study (BAsel Study on the ELderly) aiming to investigate preclinical cognitive markers of AD (see [26, 27] for details) and who constituted the reference (i.e., the normative) sample for the German version of the CERAD-NAB [28]. Baseline testing was conducted between 1997 and 2001. A detailed description of the bi-annual follow-up assessments within the BASEL study is described elsewhere [26]. Eighteen participants were excluded from the baseline sample because a detailed review of the charts at follow-up revealed that these individuals should have been excluded based on medical exclusion criteria [i.e., prostate carcinoma (*n* = 5), use of psychoactive substances (*n* = 3), loss of consciousness of more than 5 min (*n* = 2), cardiac dysfunction/aortic stenosis (*n* = 2), sensory deficits (*n* = 2), history of high temperature episode (*n* = 2), temporal arteritis (*n* = 1), or chronic pain (*n* = 1)]. Thus, the final sample consisted of 1,081 German-speaking healthy normal participants (407 women/674 men). Age ranged from 49 to 92 years (*M* = 68.6 years; SD = 7.8 years), and participants had a mean educational level of 12.5 years (SD = 3.0 years; range 7–20 years). Mini-Mental State Examination (MMSE; [29]) test scores ranged from 24 to 30, with a mean of 28.9 (SD = 1.1). To ensure healthy baseline cognitive status of all participants, the following procedure to establish the gold standard was applied: All participants were interviewed with a comprehensive medical questionnaire and were only included in the analyses, when they were cognitively healthy. Participants were excluded if they fulfilled any of the following criteria (see also [27]): (1) severe hearing, visual, or verbal deficits that interfered with the administration of neuropsychological testing; (2) severe motor deficits that interfered with everyday life; (3) severe systemic diseases (e.g., severe cardiac, renal, or liver dysfunctions, or severe endocrinological, or gastrointestinal diseases); (4) chronic pain; (5) major psychiatric disorders according to the fourth edition of the DSM (DSM-IV; [30]); (6) current use of psychoactive substances; (7) current or past diseases of the central nervous system; (8) cerebrovascular diseases (e.g., stroke, transient ischemic attack); (9) generalized atherosclerosis; and (10) diseases or events during life that could have negatively affected the central nervous system (e.g., head trauma with loss of consciousness >5 min, alcohol abuse, or general anesthesia within the last 3 months). In addition, if a participant exhibited low cognitive performance (i.e., a *z*-score ≤ −1.96 in more than one of 11 CERAD-NAB variables, including the MMSE [29] (see [27] for details) and their spouses (or another family member) reported a decline of the participant’s cognitive functions, the participant was excluded from the normative sample.

Additionally, two subsamples of this normative sample were considered to examine potential differences in the number of low scores (see “Analyses” section) already at this stage of normalcy between participants who later developed AD dementia (NC–AD) and participants who remained cognitively healthy (NC–NC). Even a marginal difference in the number of low scores between these two samples would corroborate the importance to take base rates into account. The NC–NC subsample consisted of 26 participants who remained healthy during the whole observation time and who were matched for age (±5 years), gender (exact), and education (±5 years) to the 26 participants who were healthy at baseline, but developed AD dementia years later. In addition, each NC–NC participant needed to be in the study for at least as long as his/her NC–AD counterpart. AD was diagnosed by the Memory Clinic Basel according to the National Institute of Neurological and Communicative Disorders and Stroke and the Alzheimer’s Disease-Related Disorders Association (NINCDS–ADRDA) criteria [31] and the DSM-IV [30] after referral from study coordinators due to participant—or informant-based concerns about cognitive deterioration or objective cognitive decline. Diagnosis was based on additional neuropsychological results, magnetic resonance imaging, medical and neurological examination as well as blood and serum analyses [32]. The two subgroups were comparable with respect to age, years of education, gender distribution, and MMSE score, but differed with respect to observation time such that NC–NC participants were significantly longer in the study compared to NC–AD participants (see Table 1). AD dementia was diagnosed in the NC–AD group after approximately 8 years (*M* = 8.5 years, range 3.2–13.3 years). The study was approved by the Ethics Committee of both Basel (Switzerland) and was performed in accordance with the ethical standards laid down in the 1964 Declaration of Helsinki and its later amendments. All participants gave written informed consent.

**Table 1:** Demographic characteristics and MMSE score of the two subsamples [i.e., the normal control group who remained healthy (NC–NC) and the initially healthy participants who progressed to AD dementia (NC–AD)]

	NC–NC^a^ (*n* = 26)	NC–AD^b^ (*n* = 26)	*T*/*χ* ^2^	*p* value
Age ± SD^c^ (years)	72.3 ± 5.4	72.8 ± 4.6	0.33	0.74
Education ± SD (years)	12.8 ± 2.8	12.6 ± 2.7	−0.20	0.84
%women	42.3	42.3	0.00^d^	1.00
MMSE^e^ ± SD	29.1 ± 1.0	28.7 ± 1.4	−1.15	0.26
Observation time ± SD (years)	10.8 ± 3.03	7.8 ± 2.7	−3.74	<0.001

^a^NC–NC = cognitively healthy participants who remained healthy

^b^NC–AD = initially healthy participants who progressed to Alzheimer’s disease dementia

^c^ *SD* standard deviation

^d^ *χ* ^2^ test

^e^ *MMSE* Mini-mental state examination [29]

### Neuropsychological measures

All participants were administered the German version of the CERAD-NAB [21, 22]. This battery included seven subtests measuring verbal episodic learning (Wordlist–Encoding), verbal episodic memory (Wordlist–Delayed recall and Recognition), constructional praxis (Figures–Copy), visual episodic memory (Figures–Delayed recall), executive functions (verbal fluency), and language (Boston Naming Test, 15-items). Additionally, we computed three variables: a measure reflecting the proportion of correct words recalled during verbal delayed recall relative to the words recalled at word list learning trial 3 (Wordlist–Savings), a measure representing the total number of intrusions at Wordlist–Encoding and Wordlist–Delayed recall (Wordlist–Intrusion errors), and a measure describing the proportion of correctly reproduced figures at Figures–Delayed Recall relative to Figures–Copy (Figures–Savings). A description of these tests is provided in Table 2. Altogether, ten raw scores and demographically adjusted for age, gender, and education *z*-scores were derived (see [25, 33]). Because education is a strong predictor for premorbid cognitive performance, the number of years of education was used as its surrogate [13, 34, 35]. A summary of the overall neuropsychological CERAD-NAB test performance is provided in Table S1 in the electronic Supplementary Materials (see Online Resource 1).

**Table 2:** Description of neuropsychological subtests of the CERAD-NAB^a^ [21, 22] used in this study

Test variable	Test description	Function
CERAD-NAB^a^ Wordlist–Encoding	Total number of correctly learned words across three learning trials (number of words per trial = 10)	Verbal episodic learning
CERAD-NAB^a^ Wordlist–Delayed recall	Total number of correctly remembered words after Wordlist–Encoding	Verbal episodic memory
CERAD-NAB^a^ Wordlist–Savings	Proportion correct words recalled during Wordlist–Delayed recall relative to words learned at Wordlist–Encoding learning trial 3	Verbal episodic memory
CERAD-NAB^a^ Wordlist–Discriminability	Percent of correctly recognized words from Wordlist–Encoding	Verbal episodic memory
CERAD-NAB^a^ Wordlist–Intrusion errors	Total number of intrusions at Wordlist–Encoding and Wordlist–Delayed recall	Executive functions
CERAD-NAB^a^ Figures–Copy	Copy of four figures (circle, diamond, overlapping rectangles, cube)	Constructional praxis
CERAD-NAB^a^ Figures–Delayed recall	Recall of figures reproduced at Figures–Copy	Visual episodic memory
CERAD-NAB^a^ Figures–Savings	Proportion correctly reproduced figures at Figures–Delayed recall relative to Figures–Copy	Visual episodic memory
Verbal fluency–Animals	Number of animals reproduced within 1 min	Executive functions
BNT^b^ (15-items)	Spontaneous naming of 15 black and white line drawings	Language

^a^ *CERAD-NAB* Consortium to Establish a Registry for Alzheimer’s Disease-Neuropsychological Assessment Battery

^b^ *BNT* Boston naming test

### Analyses

Pearson product moment correlations were performed on the *z*-scores of all ten CERAD-NAB variables to illustrate subtest intercorrelations. The prevalence of CERAD-NAB low scores were calculated from the overall normative sample and both subsamples for six different cut-off scores that are frequently applied in clinical practice (see, e.g., [13]). The cut-off scores are listed below.

1. 1st percentile (*z*-score ≤ −2.32).
2. 2.5th percentile (*z*-score ≤ −1.96).
3. 7th percentile (*z*-score ≤ −1.48).
4. 10th percentile (*z*-score ≤ −1.28).
5. 16th percentile (*z*-score ≤ −1.00).
6. 25th percentile (*z*-score ≤ −0.67).

Because memory impairment alone is sufficient for a diagnosis of amnestic MCI [9], an additional analysis was conducted focusing only on the CERAD-NAB memory domains. Thus, the prevalence of low CERAD-NAB scores in the verbal and visual memory was calculated: Wordlist–Encoding, Wordlist–Delayed recall, Wordlist–Discriminability, Wordlist–Savings, Wordlist–Intrusion errors, Figures–Delayed recall, and Figures–Savings. These results are reported in the electronic Supplementary Material section (see Online Resource 2). To estimate the variability of the number of low scores in the 10 CERAD-NAB variables as well as to obtain the 95 % confidence intervals (CI), we computed 1,000 bootstrap replicates [36].

In order to aid clinicians in daily practice and to facilitate clinical decision making (i.e., to determine whether a certain cognitive profile should be considered as normal or impaired), we aimed to provide a summary figure with the exact percentages of healthy older participants who obtain a certain number of low scores at or below each of the cut-off scores. Because cognitive impairment (due to any reason) is variably defined in clinical practice, we determined that probable cognitive impairment may be assumed, when <10 % of healthy older adults obtain a certain number of low scores below a given cut-off score (see also [1, 12]). That is, if the number of scores below a certain cut-off score was obtained by approximately 10 % or less participants of the normative sample, we labeled this performance as *probable cognitive impairment*. Of course, any other cutoff (e.g., 5th percentile) could be used depending on the need of the examiner (e.g., high sensitivity vs. high specificity; the 10 % cutoff used here serves as an example).

To examine whether this critical 10 % border line may detect very early and subtle signs of cognitive impairment for each of the six cut-off scores, we compared baseline neuropsychological performance of 26 healthy participants who progressed to AD dementia (NC–AD) years later and their matched control group (NC–NC). Two-sided Fisher’s exact tests were performed to determine baseline differences (i.e., at a healthy stage) in the subgroups, using an alpha level of *p* < 0.05. All statistical analyses were performed with SPSS version 21 (SPSS Inc. IBM company, 2012).

## Results

The correlation analysis between the *z*-scores of the ten CERAD-NAB tests revealed a pattern of coefficients that were mostly small to medium, indicating minimal to modest multicollinearity. Nearly all the correlations were smaller than 0.35. The largest correlations were as follows: Figures–Delayed Recall and Figures–Savings = 0.89, Wordlist–Delayed Recall and Wordlist–Savings = 0.76, Wordlist–Delayed Recall and Wordlist–Encoding = 0.648, and Wordlist–Delayed Recall and Wordlist–Recognition = 0.47.

**Fig. 1:**
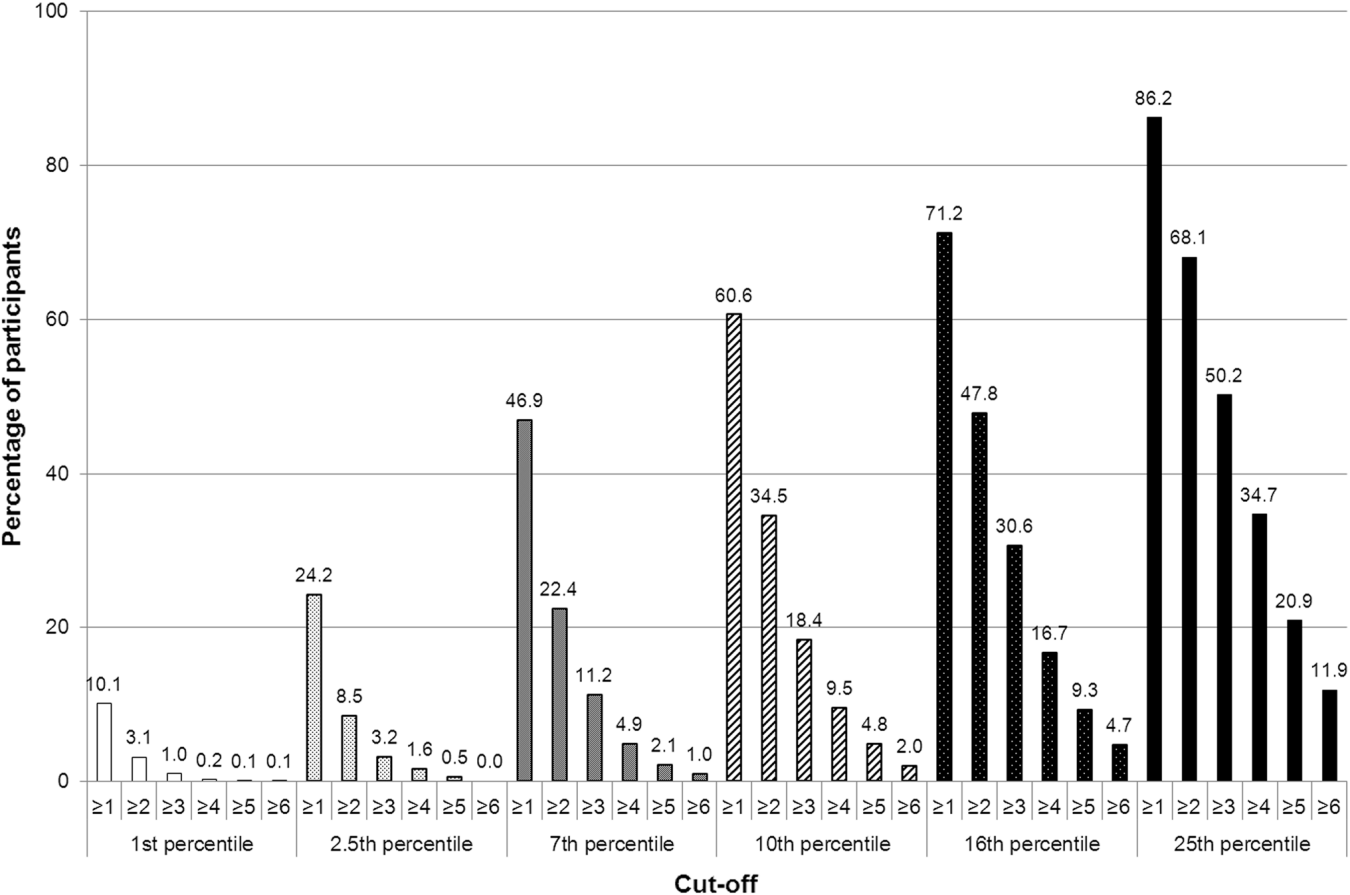
Cumulative percentages of healthy older participants with a particular minimum number of low scores in the CERAD-NAB for six different cut-off scores

Figure 1 shows the cumulative percentages of healthy individuals who have a specific number of low test scores at or below each of the six cut-off scores. Because the percentage of participants who obtain seven or more low scores was very small (i.e., ≤7.5 %) even for the less stringent cut-off scores, Fig. 1 only illustrates the percentage of participants who obtained up to six low scores (i.e., ≥1, ≥2, ≥3, ≥4, ≥5, ≥6). These data show that a high percentage of the normative sample obtains at least one or more low scores at most cut-off scores and that the number of low scores varies as a function of the cut-off score.

**Fig. 2:**
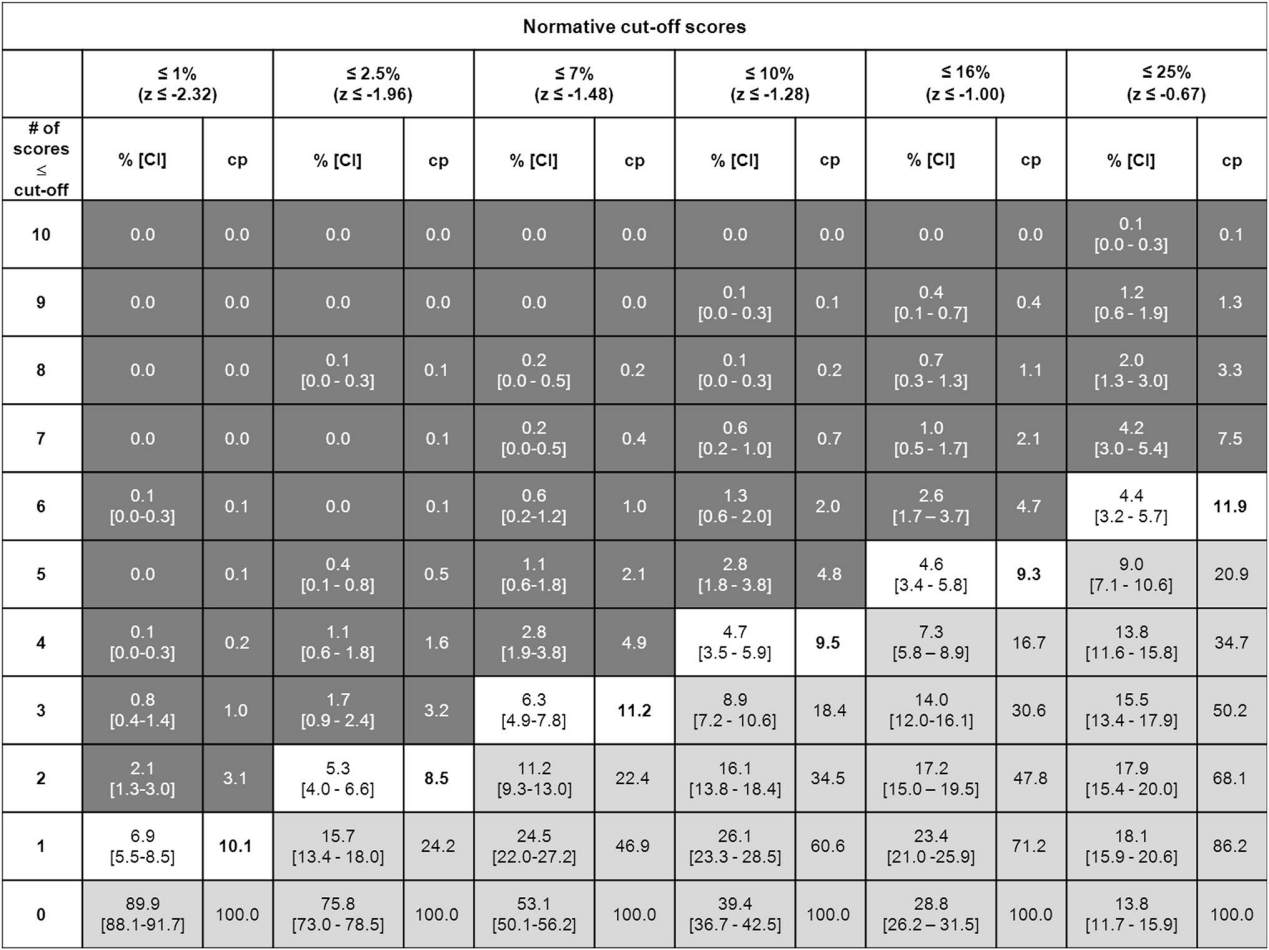
Base rates (in %) of demographically adjusted low *z*-scores out of ten CERAD-NAB variables (*far left column*) for six different cut-off scores (*second row* from the *top*). *CI* 95 % confidence interval, *cp* cumulative percentage. The *white area* represents a critical border where circa 10 % of all participants (*N* = 1,081) obtain a certain number of low scores and serves a threshold to differentiate between low (*light gray area*) and high (*dark gray area*) probabilities of pathological performance. Thus, neuropsychological results located in the *light gray area* would be interpreted as within normal limits, whereas results in the *dark gray area* would be interpreted as probable cognitive impairment

In addition to the exact percentage of healthy individuals who obtain a certain number of low scores at or below each cut-off score, Fig. 2 provides information about the number of low scores required to determine probable cognitive impairment as well as the 95 % CI calculated by using the bootstrap method [36]. Given our definition, we set the critical border, where approximately 10 % of all participants obtain a certain number of low scores (see Fig. 2; white area). This 10 % border serves as a critical threshold to differentiate between broadly normal numbers of low scores (see Fig. 2, light gray area) and an ambiguous area representing higher uncertainty about the diagnostic accuracy (see Fig. 2, dark gray area), i.e., participants whose number of low scores is situated in the light gray area are likely to be diagnosed as cognitively healthy, because a high percentage of the normative sample obtained a similar number of low scores, whereas the cognitive status of individuals whose number of low scores falls above the border in the dark gray area may be considered as abnormal, because only a small number (at most ≤7.5 % at 25th percentile, see Fig. 2 last column) of healthy older adults obtain such a high number of low scores. Thus, according to Fig. 2, when using the 10 % border as the critical threshold, probable cognitive impairment across all 10 scores would be based on obtaining one or more low scores ≤1st percentile (*z* ≤ −2.32; obtained by 10.1 % of the normative sample), two or more low scores ≤2.5th percentile (*z* ≤ −1.96; obtained by 8.5 % of the normative sample), three or more low scores ≤7th percentile (*z* ≤ −1.48; obtained by 11.2 % of the normative sample), four or more low scores ≤10th percentile (*z* ≤ −1.28; obtained by 9.5 % of the normative sample), five or more low scores ≤16th percentile (*z* ≤ −1.00; obtained by 9.3 % of the normative sample), or six or more low scores ≤25th percentile (*z* ≤ −0.67; obtained by 11.9 % of the normative sample). The results of the separate analysis with the verbal and visual episodic memory domain only are reported in the electronic Supplementary Material section (see Online Resource 2).

**Fig. 3:**
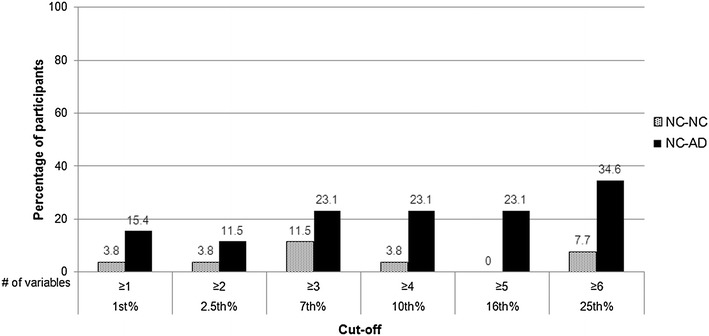
Percentage of normal controls who remained normal (NC–NC; *n* = 26) and of initially healthy participants who later obtained a diagnosis of AD dementia (NC–AD; *n* = 26) situated in the critical *dark gray area* beneath the 10 % border (see Fig. 2) at each cutoff (*x*-axis) at baseline

Figure 3 illustrates the percentage of the NC–NC and NC–AD groups situated in the critical area beneath the 10 % border (see Fig. 2) for each cut-off score at baseline examination. Consistently, more NC–AD participants are situated in the critical dark gray area compared to NC–NC participants irrespective of the used cut-off score (see Fig. 3; Table 3). Two-sided Fisher’s exact tests were performed to examine potential baseline differences of the NC–NC and NC–AD groups. These results indicate only differences for less stringent cutoffs (i.e., 25th and 16th percentile) and a statistical trend toward differences in participants who later progressed to AD dementia to be located in the critical dark gray area compared to individuals who remained healthy, for the 10th percentile (see Table 3). It has to be noted that the OR for the 16th percentile could not be calculated because the denominator was zero. For purposes of estimation, we added the value 0.5 to each cell in the contingency table. The results of the separate analysis with the verbal and visual episodic memory domain only are reported in the electronic Supplementary Material section (see Online Resource 2).

**Table 3:** Comparison of percentages of participants (NC–NC^a^, NC–AD^b^) situated in the dark gray area in Fig. 2 (at baseline)

	% in the dark gray area^c^		*p* value^d^	OR^e^ (95 % CI^f^)
	NC–NC^a^ (%)	NC–AD^b^ (%)		
25th percentile	7.7	34.6	0.04*	6.35 (1.22, 33.19)
16th percentile	0	23.1	0.02*	16.8 (0.90, 315.89)
10th percentile	3.8	23.1	0.10	7.5 (0.83, 67.49)
7th percentile	11.5	23.1	0.47	2.3 (0.51, 10.41)
2.5th percentile	3.8	11.5	0.61	3.3 (0.32, 33.62)
1st percentile	3.8	15.4	0.35	4.6 (0.47, 43.78)

* *p* value < 0.05

^a^NC–NC = cognitively healthy participants who remained healthy

^b^NC–AD = initially healthy participants who progressed to Alzheimer’s disease dementia

^c^Dark gray area = to be considered as a pathological result (see Fig. 2)

^d^Fisher’s *p* value tested by Fisher’s exact test

^e^ *OR* odds ratio

^f^ *CI* confidence interval

The final set of analyses compared a simultaneous application of all impairment-criterion cut-off scores across groups. That is, we computed the base rate in the normative sample of meeting one or more criteria for probable cognitive impairment across the ten scores when all six cut-off scores are applied simultaneously. In the entire normative sample, 22 % met criteria for cognitive impairment based on meeting one or more of the criteria in the white area in Fig. 2 (i.e., this is the percentage of people who meet criterion when all criteria are considered). In the subsample of 26 NC–NC participants, 19 % met any of the criteria, whereas 54 % of the 26 NC–AD participants met criteria for cognitive impairment based on meeting one or more of the criteria of the criteria in the white area in Fig. 2. This difference is statistically significant [*p* = 0.02, OR = 4.9, 95 % CI (1.41, 16.99)]. The same analysis was conducted for the memory domain only, and results are reported in the electronic Supplementary Material section (see Online Resource 2). Moreover, in the electronic Supplementary Material section, we also provide additional variations of combined criteria and their corresponding base rates (see Online Resource 3).

## Discussion

This study provides information about the base rates of low scores in the CERAD-NAB in a normative sample of older adults using six different cut-off scores commonly applied in clinical practice and research. The summary figure (Fig. 2) may be used as an important supplement in clinical practice when assessing cognitive performance and aims to reduce diagnostic errors in clinical decision making. Our main results support a number of studies demonstrating that a substantial percentage of healthy children [37–39], healthy adults [40, 41], and healthy older adults [1, 3, 5, 15] will obtain scores that fall within abnormal ranges when multiple tests are administered.

According to the Gaussian distribution, the number of low scores varies as a function of the cut-off score—the higher the cut-off score, the more abnormal test scores are required to diagnose cognitive impairment. However, the use of a certain cut-off score critically relates to the sensitivity and specificity of a test. For example, using a less stringent criterion for impairment [e.g., the 25th percentile (*z*-score ≤ −0.67)] indeed increases the identification of true impairment (increased sensitivity), but it is also associated with reduced specificity (i.e., the percentage of healthy individuals erroneously being classified as impaired is high; see Fig. 1) [12]. Moreover, administering more than one neuropsychological test, which is mandatory to obtain a comprehensive understanding of someone’s cognitive performance, yields multiple test scores and inherently increases the probability of abnormal scores [12, 14, 15]. For example, Schretlen et al. [15] reported that when examining healthy adults’ cognitive abilities by administering a test battery containing 10, 25 or 43 subtests, 15, 40 and 57 %, respectively, of healthy adults obtain two or more scores below the 7th percentile (i.e., *z*-score = −1.5). Moreover, choosing a more stringent criterion (*z*-score = −1.96), still 3, 13 and 24 %, respectively, obtain two or more low scores.

Beside these methodological issues, possible explanations for low test performance among these healthy adults may be due to situational or personal factors such as motivational fluctuations, increased distractibility during testing, inattentiveness, and measurement error [20], or also longstanding personal weaknesses in certain areas or intraindividual cognitive variability across different cognitive domains [5, 42]. Another explanation of low scores in cognitively normal adults is the possibility of poor effort. However, as Binder et al. [20] mention in their review—and this is very much in line with our own observations—participants taking part in research studies rarely exhibit insufficient effort, since their participation is voluntary. Moreover, systematic and random errors as additional sources for low scores were minimized as best as possible. Specifically, due to the large number of participants in this study, multiple examiners were needed to administer the tests. All examiners were thoroughly trained by experienced neuropsychologists, and instructions were given in a highly standardized way. In order to reduce any discomfort of participants, a warming-up chat was carried-out at the beginning of the examination, which took place in a quiet room without distractors. In addition, if necessary and appropriate, short breaks during the testing were allowed to avoid fatigue. To safeguard against errors at data entry, this procedure was triple-checked. Thus, these results emphasize that low scores do not necessarily need to be indicative of impairment, but may also represent normal variability of cognitive abilities within healthy adults. This is also suggested by a number of recent studies which illustrate that a considerable percentage of participants (up to 25 %) are diagnosed as cognitively impaired at baseline evaluation but revert to normal levels of functioning at follow-up examination [43–45], indicating diagnostic instability over time. Indeed, some of these misdiagnosed patients may have reverted due to adequate treatment of factors that may have caused cognitive impairment other than neurodegeneration (e.g., mood disorder, vascular risk factors). However, it can be assumed that this high number of individuals exhibiting improved or normal cognition at follow-up also contains a certain number of incorrectly diagnosed people at baseline because the probability of obtaining some low scores was not taken into account [44]. Interestingly, there is evidence that the likelihood to score within normal limits at follow-up is higher when the low scores at baseline were obtained in non-memory tests (i.e., non-amnestic MCI) [44, 45]. These results indicate that, in addition to the pure number of variables with a low score, it is important to also consider the affected cognitive domain.

The present study, however, also illustrates that some of these “healthy” participants may not be healthy and might be at a higher risk to progress to AD (i.e., they are in a prodromal stage of dementia). For example, our findings show that six or more scores (out of ten) below the 25th percentile occurred in about 12 % of the normative sample (see Fig. 2), in 8 % of the 26 older adults who remained cognitively healthy over time, but in almost 35 % of older adults who progressed to dementia (see Fig. 3). However, this result must be interpreted with caution because sample sizes were small. Nevertheless, the future dementia patients might have obtained low scores because they were already on the path of cognitive decline associated with future dementia. This thought corroborates with findings of current neuropsychological research investigating the preclinical stages of MCI and AD suggesting that low cognitive performance can be evidenced in a premorbid phase of later progressors to dementia more than a decade before diagnosis [7, 26, 46, 47]. For example, Amieva et al. [46] found in a sample of 350 preclinical AD patients and in demographically matched healthy controls that semantic and episodic memory declined 9–12 years prior to the diagnosis of AD dementia [46]. In light of these considerations, using our summary figure (Fig. 2) may help to improve diagnostic decision making in clinical practice. An illustrative example for the application of these base rates can be found in the electronic Supplementary Materials section (see Online Resource 4).

This study has some limitations to consider. The sample used in this study was a convenience sample of a subsample of the original Basel study initiated in 1959, consisting predominantly of (former) employees of the pharmaceutical industry in Basel [48]. Thus, its representativeness is somewhat limited to rather better educated older individuals. Additionally, all CERAD-NAB variables exhibit a skewed distribution, comparable to most neuropsychological test variables. However, a close approximation to a normal distribution for all neuropsychological variables was accomplished by applying monotone transformations [25]. Because we aimed to improve diagnostic accuracy in a neuropsychological assessment and low cognitive performance is indicative of impairment, normally distributed scores are primarily needed for the diagnostically relevant lower tail, while a more liberal criterion for the upper tail does not influence diagnostic validity. Further, although all participants underwent a careful and comprehensive neuropsychological and medical screening, it is not known whether some of the participants in the normative study were in a prodromal stage of AD, a condition that would mitigate the results. As illustrated in the subsample of 26 participants who later were diagnosed with dementia, we presume that some actually might have already been in a prodromal stage. The results from the baseline comparison between the NC–NC and NC–AD group are based on a very small sample and rather serve as a qualitative comparison. These results need to be studied in a larger sample. Moreover, we treated all CERAD-NAB variables as equally informative, although a high number of them were related to episodic memory.

Early and accurate diagnosis of MCI and AD represents a major and challenging goal of current neuropsychological research. The results of the present study substantially contribute to an improvement and facilitation of neuropsychological diagnostics and are highly relevant in clinical practice by illustrating that awareness of the base rates of low scores has important implications for the diagnosis of MCI or prodromal AD. It is important to note that these base rate data are meant to only supplement clinical judgment, as neuropsychological test results need to be interpreted in conjunction with results of additional examinations (e.g., the patient’s medical history and premorbid functioning (see also [19], informant-based and self-reported changes in the activities of daily living, structural magnetic resonance imaging, results cerebrospinal fluid analyses [49], etc.). Future research should investigate whether base rates of low scores in specific cognitive domains may have differential diagnostic and prognostic value. Additionally, as already mentioned, a broad range of cut-off scores is used to assess cognitive impairment. Future investigations with a large longitudinal sample might help to empirically define the optimal cut-off score(s) to discriminate between incipient neurocognitive disorder and normal aging. Moreover, because repeated neuropsychological testing is commonly used to evaluate decline over time, future research may apply the same methodology to determine the base rates of abnormal change of scores. By defining normal variability in change scores at subsequent evaluations, interpretation of follow-up results will be more valid.

## Electronic supplementary material

Below is the link to the electronic supplementary material.

Supplementary material 1 (PDF 17 kb)

Supplementary material 2 (PDF 1,015 kb)

Supplementary material 3 (PDF 26 kb)

Supplementary material 4 (PDF 74 kb)
