# Supplementary material for: Considering the base rates of low performance in cognitively healthy older adults improves the accuracy to identify neurocognitive impairment with the Consortium to Establish a Registry for Alzheimer’s Disease-Neuropsychological Assessment Battery (CERAD-NAB)
Source: Eur Arch Psychiatry Clin Neurosci. 2015 Jan 3;265(5):407–17. doi: 10.1007/s00406-014-0571-z (PMC4464368; doi:10.1007/s00406-014-0571-z)
Supplement: Supplementary file 1 — Supplementary material 1 (PDF 17 kb) [file 406_2014_571_MOESM1_ESM.pdf]

Electronic Supplementary Material 1 – Summary of overall CERAD-NAB test performance (raw scores)

European Archives of Psychiatry and Clinical Neuroscience

Considering the base rates of low performance in cognitively healthy older adults improves the accuracy to identify neurocognitive impairment with the Consortium to Establish a Registry for Alzheimer's Disease-Neuropsychological Assessment Battery (CERAD-NAB)

Panagiota Mistridis[1,2], Simone C. Egli[1,2], Grant L. Iverson[3,4], Manfred Berres[5], Klaus Willmes[6], Kathleen A. Welsh-Bohmer[7], Andreas U. Monsch[1,2*]

[1] Memory Clinic, Felix Platter Hospital, University Center for Medicine of Aging Basel, Schanzenstrassse 55, 4031 Basel, Switzerland

[2] University of Basel, Department of Psychology, Missionsstrasse 60/62, 4055 Basel, Switzerland

[3] Department of Physical Medicine and Rehabilitation, Harvard Medical School

[4] Red Sox Foundation and Massachusetts General Hospital Home Base Program, Boston, MA 02114, USA

[5] Department of Mathematics and Technology, University of Applied Sciences Koblenz, Joseph-Rovan-Allee 2, 53424 Remagen, Germany

[6] Section Neuropsychology, Department of Neurology, RWTH Aachen University, Pauwelsstraße 30, 52074 Aachen, Germany

[7] Joseph and Kathleen Bryan Alzheimer's Disease Center, Duke University, 2200W Main Street, Suite A200, Durham NC 27705, USA

*Correspondence concerning this article should be addressed to:

Andreas U. Monsch, Memory Clinic, Felix Platter Hospital, University Center for Medicine of Aging Basel, Schanzenstrasse 55, 4031 Basel, Switzerland

Phone: +41 61 265 31 93

Fax: +41 61 265 37 94

E-Mail: Andreas.Monsch@unibas.ch

Table S1 Descriptive statistics for the raw scores of the 10 CERAD-NAB[a] [21, 22] measures (*N* = 1,081)

	M (SD)	Median	IQR[b]
Verbal Fluency	21.4 (5.6)	21.0	8.0 (17, 25)
Boston Naming Test [15]	14.0 (1.1)	14.0	2.0 (15, 13)
Word list - Encoding [30]	20.5 (3.6)	21.0	5.0 (23, 18)
Word list - Delayed Recall [10]	7.0 (2.0)	7.0	2.0 (6, 8)
Word list - Discriminability (%) [100]	96.9 (4.9)	100.0	5.0 (95, 100)
Word list - Savings (%)	85.9 (19.9)	87.5	25.0 (75, 100)
Word List - Intrusion Errors	0.7 (1.3)	0.0	1.0 (0, 1)
Figures - Copy [11]	10.3 (0.9)	11.0	1.0 (10, 11)
Figures - Delayed Recall [11]	8.9 (2.1)	10.0	4.0 (7, 11)
Figures - Savings (%)	85.7 (19.4)	91.9	28.3 (72.7, 100)

Maximal scores are given in square brackets

[a] CERAD-NAB = Consortium to Establish a Registry for Alzheimer's Disease-Neuropsychological Assessment Battery

[b] IQR = interquartile range
