# Supplementary material for: Considering the base rates of low performance in cognitively healthy older adults improves the accuracy to identify neurocognitive impairment with the Consortium to Establish a Registry for Alzheimer’s Disease-Neuropsychological Assessment Battery (CERAD-NAB)
Source: Eur Arch Psychiatry Clin Neurosci. 2015 Jan 3;265(5):407–17. doi: 10.1007/s00406-014-0571-z (PMC4464368; doi:10.1007/s00406-014-0571-z)
Supplement: Supplementary file 2 — Supplementary material 2 (PDF 1,015 kb) [file 406_2014_571_MOESM2_ESM.pdf]

Electronic Supplementary Material 2 - Base rates of low scores for the verbal and visual CERAD-NAB episodic memory domain

European Archives of Psychiatry and Clinical Neuroscience

Considering the base rates of low performance in cognitively healthy older adults improves the accuracy to identify neurocognitive impairment with the Consortium to Establish a Registry for Alzheimer's Disease-Neuropsychological Assessment Battery (CERAD-NAB)

Panagiota Mistridis[1,2], Simone C. Egli[1,2], Grant L. Iverson[3,4], Manfred Berres[5], Klaus Willmes[6], Kathleen A. Welsh-Bohmer[7], Andreas U. Monsch[1,2*]

[1] Memory Clinic, Felix Platter Hospital, University Center for Medicine of Aging Basel, Schanzenstrassse 55, 4031 Basel, Switzerland

[2] University of Basel, Department of Psychology, Missionsstrasse 60/62, 4055 Basel, Switzerland

[3] Department of Physical Medicine and Rehabilitation, Harvard Medical School

[4] Red Sox Foundation and Massachusetts General Hospital Home Base Program, Boston, MA 02114, USA

[5] Department of Mathematics and Technology, University of Applied Sciences Koblenz, Joseph-Rovan-Allee 2, 53424 Remagen, Germany

[6] Section Neuropsychology, Department of Neurology, RWTH Aachen University, Pauwelsstraße 30, 52074 Aachen, Germany

[7] Joseph and Kathleen Bryan Alzheimer’s Disease Center, Duke University, 2200W Main Street, Suite A200, Durham NC 27705, USA

*Correspondence concerning this article should be addressed to:

Andreas U. Monsch, Memory Clinic, Felix Platter Hospital, University Center for Medicine of Aging Basel, Schanzenstrasse 55, 4031 Basel, Switzerland

Phone: +41 61 265 31 93

Fax: +41 61 265 37 94

E-Mail: Andreas.Monsch@unibas.ch

Base rates of Low Scores for the Verbal and Visual CERAD-NAB

Episodic Memory Domain

The prevalence of low scores for the CERAD-NAB verbal and visual episodic memory domain was calculated from the overall normative sample and both subsamples for six different cut-off scores that are frequently applied in clinical practice [1]. The cut-off scores are listed below.

(1) 1st percentile (z-score ≤ -2.32)

(2) 2.5th percentile (z-score ≤ -1.96)

(3) 7th percentile (z-score ≤ -1.48)

(4) 10th percentile (z-score ≤ -1.28)

(5) 16th percentile (z-score ≤ -1.00)

(6) 25th percentile (z-score ≤ -0.67)

According to the analyses including all ten CERAD-NAB variables, we conducted the same analyses with only the seven CERAD-NAB memory variables (i.e., Wordlist–Encoding, Wordlist–Delayed recall, Wordlist–Discriminability, Wordlist–Savings, Wordlist–Intrusion errors, Figures–Delayed recall, and Figures–Savings). To estimate the variability of the number of low scores in the seven CERAD-NAB memory variables as well as to obtain the 95 % confidence intervals (CI), we computed 1,000 bootstrap replicates [2].

Results

The results of the additional analyses including only the verbal and visual episodic memory domain are presented in Fig. S1. Given our definition, we set the critical border, where approximately 10 % of all participants obtain a certain number of low scores (see Fig. S1; white area). This 10%-border serves as a critical threshold to differentiate between broadly normal numbers of low scores (see Fig. S1, light gray area) and an ambiguous area representing higher uncertainty about the diagnostic accuracy (see Fig. S1, dark gray area), i.e., participants whose number of low scores is situated in the light gray area are likely to be diagnosed as cognitively healthy, because a high percentage of the normative sample obtained a similar number of low scores, whereas the cognitive status of individuals whose number of low scores falls above the border in the dark gray area may be considered as abnormal, because only a small number (at most 5.8 % at 25th percentile, see Fig. S1 last column) of healthy older adults obtain such a high number of low scores. Thus, according to Fig. S1, when

using 10 %-border as the critical threshold, probable memory impairment across all seven scores would be based on obtaining one or more low memory scores ≤ 1st percentile (*z* ≤ -2.32; obtained by 7.7 % of the normative sample), two or more low memory scores ≤ 2.5th percentile (*z* ≤ -1.96; obtained by 6.8 % of the normative sample), three or more low memory scores ≤ 7th percentile (*z* ≤ -1.48; obtained by 7.1 % of the normative sample), three or more low memory scores ≤ 10th percentile (*z* ≤ -1.28; obtained by 11.2 % of the normative sample), four or more low memory scores ≤ 16th percentile (*z* ≤ -1.00; obtained by 10.5 % of the normative sample), or five or more low memory scores ≤ 25th percentile (*z* ≤ -0.67; obtained by 11.0 % of the normative sample).

Normative cut-off scores												
	≤ 1% (z ≤ -2.32)		≤ 2.5% (z ≤ -1.96)		≤ 7% (z ≤ -1.48)		≤ 10% (z ≤ -1.28)		≤ 16% (z ≤ -1.00)		≤ 25% (z ≤ -0.67)	
# of scores ≤ cut-off	% [CI]	cp	% [CI]	cp	% [CI]	cp	% [CI]	cp	% [CI]	cp	% [CI]	cp
7	0.0	0.0	0.1 [0.0 - 0.3]	0.1	0.1 [0.0 - 0.3]	0.1	0.1 [0.0 - 0.3]	0.1	0.5 [0.2 - 1.0]	0.6	1.8 [1.0 - 2.5]	1.8
6	0.0	0.0	0.0	0.1	0.4 [0.1 - 0.8]	0.5	0.6 [0.3 - 1.2]	0.7	1.6 [0.8 - 2.3]	2.1	3.9 [2.8 - 5.1]	5.6
5	0.1 [0.0 - 0.3]	0.1	0.1 [0.0 - 0.3]	0.2	0.6 [19.3 – 24.3]	1.1	1.7 [0.9 - 2.5]	2.4	3.2 [2.2 - 4.4]	5.4	5.4 [4.1 - 6.8]	11.0
4	0.1 [0.0 - 0.3]	0.2	0.8 [0.3 - 1.5]	1.0	2.2 [60.6 – 66.1]	3.3	3.6 [2.5 - 4.8]	6.0	5.2 [4.0 - 6.6]	10.5	10.2 [8.5 - 11.9]	21.2
3	0.6 [0.2 - 1.1]	0.8	1.4 [0.7 - 2.1]	2.4	3.8 [2.7 - 5.0]	7.1	5.2 [4.0 - 6.6]	11.2	10.6 [8.7 - 12.6]	21.2	13.4 [11.5 - 15.6]	34.6
2	1.9 [1.0 - 2.7]	2.7	4.4 [3.3 - 5.7]	6.8	11.4 [9.5 - 13.3]	18.5	15.5 [13.4 - 17.6]	26.7	15.4 [13.3 - 17.4]	36.6	16.9 [14.7 - 19.3]	51.5
1	5.0 [3.7 - 6.4]	7.7	14.2 [12.1 - 16.4]	21.0	18.2 [16.0 - 20.6]	36.7	18.5 [16.3 - 20.3]	45.2	19.4 [17.0 - 21.8]	56.1	21.2 [18.6 - 23.5]	72.7
0	92.3 [90.6 - 93.8]	100.0	79.0 [76.5 - 81.4]	100.0	63.3 [60.3 - 66.2]	100.0	54.8 [51.9 - 57.8]	100.0	43.9 [41.2 - 46.9]	100.0	27.3 [24.7 - 29.8]	100.0

Fig. S1 Base rates (in %) of demographically adjusted low *z*-scores out of seven CERAD-NAB memory variables (*far left column*) for six different cut-off scores (*second row from the top*). *CI* 95 % confidence interval, *cp* cumulative percentage. The white area represents a critical border where circa 10 % of all participants (*N* = 1,081) obtain a certain number of low scores and serves a threshold to differentiate between low (*light gray area*) and high (*dark gray area*) probabilities of pathological performance. Thus, neuropsychological results located in the light gray area would be interpreted as *within normal* limits, whereas results in the dark gray area would be interpreted as *probable cognitive impairment.*

Figure S2 illustrates the percentage of the NC–NC and NC–AD groups situated in the critical area beneath the 10 % border (see Fig. S1) for each cut-off score at baseline examination when only the seven CERAD-NAB memory scores are considered.

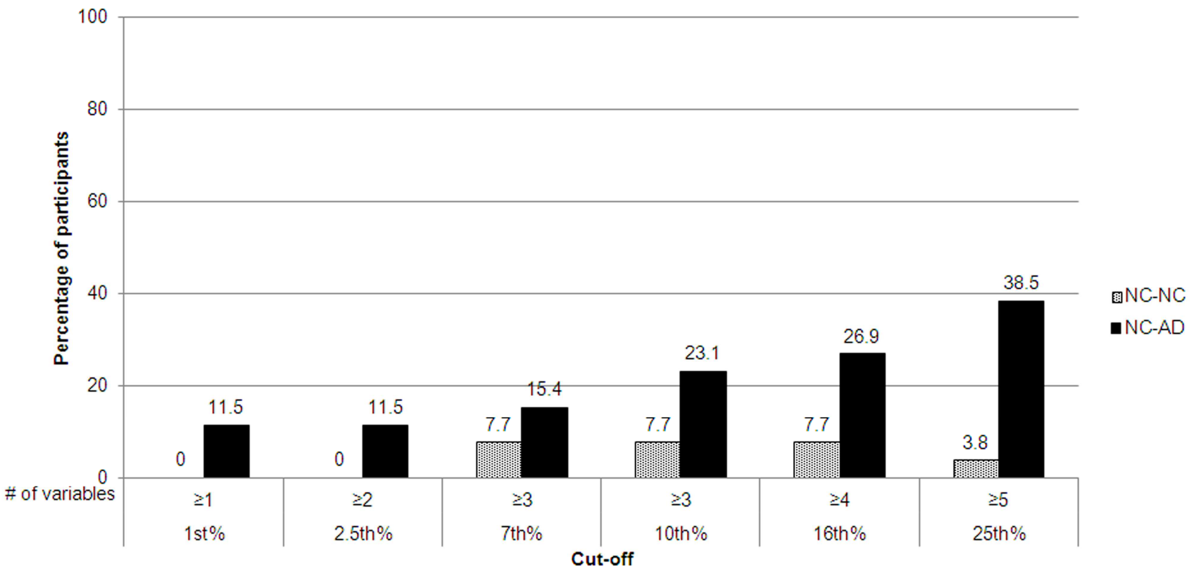

Fig. S2 Percentage of normal controls who remained normal (NC–NC; *n* = 26) and of initially healthy participants who later obtained a diagnosis of AD dementia (NC–AD; *n* = 26) situated in the critical dark gray area beneath the 10 % border (see Fig. 2) at each cutoff (*x*-axis) at baseline (number of CERAD-NAB memory tests = 7)

Consistently, more NC–AD participants are situated in the critical dark gray area compared to NC–NC participants irrespective of the used cut-of score (see Fig. S2 and Table S2). Two-sided Fisher's exact tests were performed to examine potential baseline differences of the NC–NC and NC–AD groups. These results indicate only significant results for less stringent cut-offs (i.e., 25th percentile) and a trend toward differences in participants who later progressed to AD dementia to be located in the critical dark gray area compared to individuals who remained healthy, for the 16th percentile (see Table S2).

Table S2 Comparison of percentages of participants (NC–NC[a], NC–AD[b]) situated in the dark gray area in Figure 2 (at baseline).

	**% in the dark gray area[c]**			
	**NC-NC[a]**	**NC-AD[b]**	***p* value[d]**	**OR[e] [95%CI[f]]**
25th percentile	3.8%	38.5%	0.005*	15.6 [1.82, 134.04]
16th percentile	7.7%	26.9%	0.14	4.4 [0.82, 23.79]
10th percentile	7.7%	23.1%	0.25	3.6 [0.65, 19.84]
7th percentile	7.7%	15.4%	0.67	2.2 [0.36, 13.11]
2.5th percentile	0%	11.5%	0.23	7.9 [0.39, 160.92]
1st percentile	0%	11.4%	0.24	7.9 [0.39, 16.92]

[a] NC–NC = cognitively healthy participants who remained healthy

[b] NC–AD = initially healthy participants who progressed to Alzheimer's disease dementia

[c] Dark gray area = to be considered as a pathological result (see Figure 2)

[d] Fisher's *p* value tested by Fisher's exact test

[e] OR = odds ratio

[f] CI = confidence interval

*p value < 0.05

The final set of analyses compared a simultaneous application of all impairment-criterion cut-off scores across groups. That is, we computed the base rate in the normative sample of meeting one or more criteria for probable cognitive impairment across the seven memory scores when all six cut-off scores are applied simultaneously. In the entire normative sample, 19 % met criteria for memory impairment based on meeting one or more of the criteria in the white area in Fig. S1. In the subsample of 26 NC–NC participants 8 % met criteria for memory impairment whereas 46 % of the 26 NC–AD participants met criteria for memory impairment based on meeting one or more of the criterion in the white area in Figure S1.

References

1. Lezak MD, Howieson DB, Bigler ED, Tranel D (2012) Neuropsychological assessment, 5th edn. Oxford University Press, New York

2. Efron B (1979) Bootstrap Methods: another Look at the Jackknife. Ann Stat 7(1):1–26. doi:10.1214/aos/1176344552
