# Supplementary material for: Considering the base rates of low performance in cognitively healthy older adults improves the accuracy to identify neurocognitive impairment with the Consortium to Establish a Registry for Alzheimer’s Disease-Neuropsychological Assessment Battery (CERAD-NAB)
Source: Eur Arch Psychiatry Clin Neurosci. 2015 Jan 3;265(5):407–17. doi: 10.1007/s00406-014-0571-z (PMC4464368; doi:10.1007/s00406-014-0571-z)
Supplement: Supplementary file 3 — Supplementary material 3 (PDF 26 kb) [file 406_2014_571_MOESM3_ESM.pdf]

Electronic Supplementary Material 3 – Base rates for 20 operational criteria for probable cognitive impairment

European Archives of Psychiatry and Clinical Neuroscience

Considering the base rates of low performance in cognitively healthy older adults improves the accuracy to identify neurocognitive impairment with the Consortium to Establish a Registry for Alzheimer's Disease-Neuropsychological Assessment Battery (CERAD-NAB)

Panagiota Mistridis[1,2], Simone C. Egli[1,2], Grant L. Iverson[3,4], Manfred Berres[5], Klaus Willmes[6], Kathleen A. Welsh-Bohmer[7], Andreas U. Monsch[1,2*]

[1] Memory Clinic, Felix Platter Hospital, University Center for Medicine of Aging Basel, Schanzenstrassse 55, 4031 Basel, Switzerland

[2] University of Basel, Department of Psychology, Missionsstrasse 60/62, 4055 Basel, Switzerland

[3] Department of Physical Medicine and Rehabilitation, Harvard Medical School

[4] Red Sox Foundation and Massachusetts General Hospital Home Base Program, Boston, MA 02114, USA

[5] Department of Mathematics and Technology, University of Applied Sciences Koblenz, Joseph-Rovan-Allee 2, 53424 Remagen, Germany

[6] Section Neuropsychology, Department of Neurology, RWTH Aachen University, Pauwelsstraße 30, 52074 Aachen, Germany

[7] Joseph and Kathleen Bryan Alzheimer’s Disease Center, Duke University, 2200W Main Street, Suite A200, Durham NC 27705, USA

*Correspondence concerning this article should be addressed to:

Andreas U. Monsch, Memory Clinic, Felix Platter Hospital, University Center for Medicine of Aging Basel, Schanzenstrasse 55, 4031 Basel, Switzerland

Phone: +41 61 265 31 93

Fax: +41 61 265 37 94

E-Mail: Andreas.Monsch@unibas.ch

Table S3 Base rates for 20 operational criteria for probable cognitive impairment on the CERAD-NAB[a] [1,2]

		Base rates		
**Criterion number**	**Criterion for cognitive impairment: Based on 10 Scores**	**normative sample (*N* = 1,081)**	**NC-NC[c] (*n* = 26)**	**NC-AD[d] (*n* = 26)**
1	≤ 25th percentile: 6 or more scores	11.9 %	7.7 %	34.6 %
2	≤ 16th percentile: 5 or more scores	9.3 %	0 %	23.1 %
3	≤ 10th percentile: 4 or more scores	9.5 %	3.8 %	23.1 %
4	≤ 7th percentile: 3 or more scores	11.2 %	11.5 %	23.1 %
5	≤ 2.5th percentile: 2 or more scores	8.5 %	3.8 %	11.5 %
6	≤ 1st percentile: 1 or more scores	10.1 %	3.8 %	15.4 %
7	Criteria 1-6 simultaneously: 1 or more	22.0 %	19.2 %	53.8 %
8	Criteria 1-6 simultaneously: 2 or more	13.8 %	7.7 %	26.9 %
9	Criteria #1 OR #4	16.0 %	15.4 %	42.3 %
	**Criterion for memory impairment: Based on 7 Scores**			
10	≤ 25th percentile: 5 or more scores	11.0 %	3.8 %	38.5 %
11	≤ 16th percentile: 4 or more scores	10.5 %	7.7 %	26.9 %
12	≤ 10th percentile: 3 or more scores	11.2 %	7.7 %	23.1 %
13	≤ 7th percentile: 3 or more scores	7.1 %	7.7 %	15.4 %
14	≤ 2.5th percentile: 2 or more scores	6.8 %	0.0 %	11.5 %
15	≤ 1st percentile: 1 or more scores	7.7 %	0.0 %	11.5 %
16	Criteria 1-6 simultaneously: 1 or more	19.3 %	7.7 %	46.2 %
17	Criteria 1-6 simultaneously: 2 or more	12.6 %	7.7 %	30.8 %
18	Criteria #10 OR #13	13.0 %	7.7 %	42.3 %
	**Combined criteria for probable cognitive impairment**			
19	(Verbal Fluency OR BNT[b] ≤ 16th percentile) AND #16	6.1%	0.0%	7.7%
20	Figures Copy ≤ 16th percentile AND #16	3.1	0.0%	11.5%

The base rates for each criterion (1-20) are provided for the normative sample and for two subgroups (NC–NC, NC–AD) with known future outcomes. If a single criterion is applied in research or in clinical practice, then the

associated base rate is accurate (e.g., #13 = 7.1 % in the normative sample). However, if multiple criteria are applied simultaneously, the base rates increase (e.g., see #7 and #16)

[a] CERAD-NAB = Consortium to Establish a Registry for Alzheimer's Disease-Neuropsychological Assessment Battery

[b] BNT = Boston Naming Test

[c] NC-NC = cognitively healthy participants who remained healthy

[d] NC-AD = initially healthy participants who progressed to Alzheimer’s disease dementia
