# Supplementary material for: Considering the base rates of low performance in cognitively healthy older adults improves the accuracy to identify neurocognitive impairment with the Consortium to Establish a Registry for Alzheimer’s Disease-Neuropsychological Assessment Battery (CERAD-NAB)
Source: Eur Arch Psychiatry Clin Neurosci. 2015 Jan 3;265(5):407–17. doi: 10.1007/s00406-014-0571-z (PMC4464368; doi:10.1007/s00406-014-0571-z)
Supplement: Supplementary file 4 — Supplementary material 4 (PDF 74 kb) [file 406_2014_571_MOESM4_ESM.pdf]

Electronic Supplementary Material 4 - Illustrative example for the application of the base rates

European Archives of Psychiatry and Clinical Neuroscience

Considering the base rates of low performance in cognitively healthy older adults improves the accuracy to identify neurocognitive impairment with the Consortium to Establish a Registry for Alzheimer's Disease-Neuropsychological Assessment Battery (CERAD-NAB)

Panagiota Mistridis[1,2], Simone C. Egli[1,2], Grant L. Iverson[3,4], Manfred Berres[5], Klaus Willmes[6], Kathleen A. Welsh-Bohmer[7], Andreas U. Monsch[1,2*]

[1] Memory Clinic, Felix Platter Hospital, University Center for Medicine of Aging Basel, Schanzenstrassse 55, 4031 Basel, Switzerland

[2] University of Basel, Department of Psychology, Missionsstrasse 60/62, 4055 Basel, Switzerland

[3] Department of Physical Medicine and Rehabilitation, Harvard Medical School

[4] Red Sox Foundation and Massachusetts General Hospital Home Base Program, Boston, MA 02114, USA

[5] Department of Mathematics and Technology, University of Applied Sciences Koblenz, Joseph-Rovan-Allee 2, 53424 Remagen, Germany

[6] Section Neuropsychology, Department of Neurology, RWTH Aachen University, Pauwelsstraße 30, 52074 Aachen, Germany

[7] Joseph and Kathleen Bryan Alzheimer's Disease Center, Duke University, 2200W Main Street, Suite A200, Durham NC 27705, USA

*Correspondence concerning this article should be addressed to:

Andreas U. Monsch, Memory Clinic, Felix Platter Hospital, University Center for Medicine of Aging Basel, Schanzenstrasse 55, 4031 Basel, Switzerland

Phone: +41 61 265 31 93

Fax: +41 61 265 37 94

E-Mail: Andreas.Monsch@unibas.ch

Illustrative example for the application of the base rates (see Fig. 2)

To illustrate the application of these base rates and Fig. 2, a 73-year old man with 12 years of education was administered the CERAD-NAB. His results (i.e., demographically adjusted *z*-scores) show that 3 of 10 scores were below $z = -1.28$. Because 18.4 % of the normative sample had 3 or more of the 10 variables below $z = -1.28$ (see Fig. 2), the patient's performance would be considered to be *within normal* limits. However, if the patient would have had 5 of 10 scores below $z = -1.28$, his performance would have been considered to reflect *probable cognitive impairment*, because only 4.8 % (see Fig. 2) of the normative sample performed worse. Thus, watchful waiting and a follow-up examination within the following years would be critical for this patient to ensure cognitive stability.

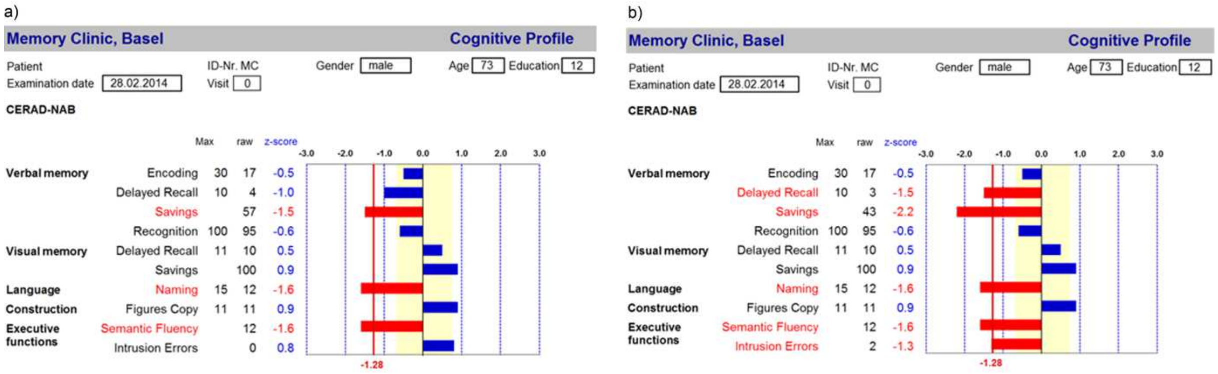

Fig. S3 Hypothetical CERAD-NAB profiles (demographically adjusted *z*-scores) of a 73-year old patient obtaining a) three low scores or b) five low scores when using a cut-off score of $z = -1.28$. Incorporating the base rates (see Fig. 2), situation a) would be interpreted as *within normal limits* (base rate = 18.4 %; Fig. 2), whereas situation b) would be judged as *probable cognitive impairment* (base rate = 4.8 %; Fig. 2). The shaded yellow area represents the 25th percentile ($z = \pm 0.67$)
